# Bayesian Analysis of Frailty Risk Factors in Chronic Kidney Disease: A Nationwide Cross‐Sectional Survey

**DOI:** 10.1002/hsr2.71698

**Published:** 2025-12-29

**Authors:** Jiao Wang, Quan Wang, Zian Zhao, Minxin Chen, Dexter Siu‐Hong Wong, James Chung‐Wai Cheung, Duo Wai‐Chi Wong

**Affiliations:** ^1^ Department of Biomedical Engineering, Faculty of Engineering The Hong Kong Polytechnic University Hong Kong SAR China; ^2^ Department of Clinical Laboratory Hubei Provincial Hospital of Traditional Chinese Medicine Wuhan China; ^3^ School of Medicine and Pharmacy Ocean University of China Qingdao China; ^4^ Research Institute for Smart Ageing The Hong Kong Polytechnic University Hong Kong SAR China

**Keywords:** CHARLS, chronic kidney disease, creatinine, cystatin C, depression, frailty

## Abstract

**Introduction:**

Frailty in chronic kidney disease (CKD) patients is associated with increased risk of adverse health outcomes. Understanding the contributing factors to frailty in this population is crucial for developing targeted interventions and improving patient care. The objective of this study is to identify and quantify potential risk factors associated with frailty in chronic kidney disease patients.

**Methods:**

We conducted a cross‐sectional study using data from the China Health and Retirement Longitudinal Study (CHARLS) cohorts of 2011 and 2015. A Bayesian mixed‐effects logistic regression model was utilized to analyze the relationship between selected features and frailty in chronic kidney disease.

**Results:**

Of 1,924 participants, about one‐third (*n* = 634) were classified as frail. Depression emerged as the strongest predictor of frailty (adjusted OR 289.49, 95%CrI 47.24–2284.82). Other significant factors included stroke (adjusted OR 18.04, 95%CrI 1.94–183.99), chronic lung disease (adjusted OR 7.96, 95%CrI 1.84–39.44), and rheumatism (adjusted OR 3.84, 95%CrI 1.12–15.14). Poor vision (adjusted OR 5.84, 95% CrI 1.68–25.25) and poor sleep quality (adjusted OR 5.12, 95% CrI 1.53–21.13), though the latter showed some instability after data imputation, were also associated with higher frailty risk. Elevated cystatin C levels showed a strong positive association (adjusted OR 52.46, 95%CrI 9.51–356.12), while higher creatinine levels were associated with lower frailty risk (adjusted OR 0.24, 95%CrI 0.07–0.81).

**Conclusion:**

This study identified several potential contributing factors to frailty in CKD patients, with depression emerging as the strongest predictor. The counterintuitive relationship between creatinine levels and frailty underscores the complex interplay between muscle mass/quality and kidney function in frailty development, warranting further investigation.

## 1 Introduction

Chronic kidney disease (CKD) is defined by a progressive decline in kidney function over time, culminating in various complications, such as cardiovascular disease, anaemia, and bone disorder. It represents a significant public health burden over one‐tenth of the global population [1]. In fact, the increasing prevalence of CKD, alongside the rising incidence of end‐stage renal failure requiring renal replacement therapy, is approaching epidemic levels [2]. Alarmingly, only a limited number of countries possess the economic capability necessary to effectively address the multifaceted challenges [3].

Among CKD complications, frailty has emerged as a particularly pressing concern. The physiological alterations associated with kidney disease can predispose individuals to frailty—a syndrome characterized by decreased physiological reserve and increased vulnerability to adverse outcomes [4]. More than 40% of CKD patients are identified as frail or prefrail [5]. Those awaiting kidney transplantation tend to be frailer, and this increased frailty negatively impacts transplant outcomes [6]. CKD patients with frailty experience a loss of appetite, tiredness, feeling cool and poor concentration [7]. They have more than twice the risk of mortality [8], as well as an increased risk of cardiovascular events, disability, falls, and hospitalization [5, 9].

Early identification of frailty in CKD can facilitate timely intervention, such as nutrition support, rehabilitation, and medical management, to mitigate progression and improve prognosis [6]. The Fried Frailty Phenotype and the Clinical Frailty Scale are commonly used to assess frailty. However, these instruments are subjective and might prone to bias, in addition to variations in the interpretation of questionnaire items [10]. Their properties were not validated in CKD patients and might not fully account for the multifactorial nature specific to CKD [10]. These methods could only classify patients into frail, pre‐fail, and not frail, thus failing to assess the severity of frailty [11]. On the other hand, physical assessments, such as walking gait and grip strength, have demonstrated higher discriminative power but might not be practical in clinical settings [12]. The findings of physical assessment might not align well with validated questionnaire instruments. There are variations in frailty criteria among physical assessments [13], which are further confounded by the type of population and other health factors [14, 15].

The availability of extensive big data repositories, such as the National Health and Nutrition Examination Survey (NHANES) and the China Health and Retirement Longitudinal Study (CHARLS), presents significant opportunities for assessing frailty risk in CKD patients. They provide rich longitudinal health data to estimate the complex patterns associated with frailty. Advanced statistical techniques and machine learning models can then be utilized to revolutionize and individualize frailty risk assessment.

The objective of this study is to identify and quantify the risk factors associated with frailty in CKD patients using a Bayesian model applied to the nationwide cross‐sectional CHARLS public dataset. We seek to determine the odds ratios of various factors potentially contributing to frailty in the CKD population. The findings from this study could enhancing our understanding of their relationship, potentially inform clinical practice by enabling earlier interventions and improving patient outcomes in the management of frailty among CKD patients.

## 2 Methods

### 2.1 Ethical Approval

This study was approved by the institutional review broad of the Hong Kong Polytechnic University (Reference number: HSEARS20251128005).

### 2.2 Data Collection and Processing

Data were collected from a community‐based, nationwide representative cohort study, the China Health and Retirement Longitudinal Study (CHARLS), which focuses on Chinese adults [16]. We extracted data from the 2011 and 2015 cohort due to its inclusion of blood test data. The CHARLS dataset comprised samples from 150 counties across 28 provinces in China, utilizing a stratified multistage cluster sampling method. The CHARLS survey received prior approval from Peking University and received informed consent from each subject. Figure S1 shows a flowchart on participant selection and the overall workflow.

We included data only from individuals with chronic kidney disease. The presence of chronic kidney disease was identified either through self‐reported medical history or by an estimated glomerular filtration rate (eGFR) of less than 60 mL per minute per 1.73 m^2^ body surface area [17]. eGFR (per min per 1.73 m^2^ body surface area) was calculated based on the Collaborative Epidemiological Study of Chronic Kidney Disease (CKD‐EPI) equation [17], as shown in Equation (1).

(1) $$\mathrm{eGFR}=141\times \min{\left(\frac{\mathrm{Scr}}{\kappa },1\right)}^{\alpha }\times \max{\left(\frac{\mathrm{Scr}}{\kappa },1\right)}^{-1.209}\times 0.993^{\mathrm{Age}}\times 1.018(\mathrm{Gender}=\mathrm{Female})$$

where *Scr* is the serum creatinine concentration (in mg/dL). *κ* and *α* are gender‐specific coefficient, where the coefficient *κ* is 0.7 for female and 0.9 for male; *α* is −0.329 for female and −0.411 for male.

Samples with missing data for age, gender, or any blood test results were excluded from the analysis. We employed an imputation strategy for body height and weight based on age and gender data after the data collection. To minimize the impact of imputed data on the feature selection and reduction processes, we deferred the imputation of other missing variables until after these steps were completed (Table S2). For all imputation procedures, we utilized the multivariate imputation by chained equations (MICE) [18].

### 2.3 Frailty Outcome

Frailty was assessed using a modified version of the Fried frailty phenotype [11], adapted for the CHARLS dataset following the approach proposed by Bu, et al. [19]. While the original Fried criteria define frailty as meeting ≥ 3 out of 5 criteria, we employed a modified approach that defines frailty as meeting at least half of the available criteria, adjusting the threshold proportionally when data for certain criteria were missing.

- Criterion 1: Weight loss. An individual was considered positive in this criterion if their BMI is less than 18.5 kg/m².
- Criterion 2: Low physical activity. An individual engaged in less than 10 min of walking and moderate level of physical exercise per week.
- Criterion 3: Exhaustion. Individuals who reported that they found everything to be “effortful” and could not get “going”, more often than rarely, were classified as experiencing exhaustion.
- Criterion 4: Weakness. An individual was considered weak if they reported difficulty lifting or carrying weights greater than 5 kg.
- Criterion 5: Slowness. Individuals who faced challenges in walking 100 meters or climbing several flights of stairs without rest are identified as positive in this criterion.

### 2.4 Risk Factors (Predictors)

The CHARLS dataset encompassed several domains of data collection, including demographics, family structure and transfers, health status and functioning, biomarkers, health care and insurance, work retirement and pension, income consumption and assets, as well as life style and behaviours [16]. Given the extensive range of features available, we first selected the relevant variables (risk factors) guided by our expertise and in reference to existing literature [20, 21, 22, 23, 24].

We pre‐selected a set of 37 relevant features (risk factors). These encompassed eight demographic and behavioural factors: gender, age, body mass index (BMI), waist circumference, history of smoking, alcohol consumption, participation in social activities, and sleep quality. Additionally, we included 13 self‐reported medical conditions: hypertension, dyslipidaemia, diabetes, cancer, chronic lung disease, stroke, digestive disease, psychiatric problems, rheumatism (including arthritis), poor hearing, poor vision, pain, and depression. Besides, we included 16 blood test results: C‐reactive protein (CRP), creatinine, white blood cell count, haemoglobin, haematocrit, mean corpuscular volume (MCV), platelet count, triglycerides, blood urea nitrogen (BUN), uric acid, glycated haemoglobin, high‐density lipoprotein (HDL), low‐density lipoprotein (LDL), total cholesterol (TC), glucose, and cystatin C. It is noteworthy that history of smoking, alcohol consumption, participation in social activities, and all self‐reported medical conditions were treated as binary variables, while sleep quality was represented as a 4‐level ordinal variable with 3 points denoted “restless most or all of the time”.

### 2.5 Feature Selection/Reduction

In the feature selection or reduction phase, we employed the Boruta algorithm to identify the most relevant predictors for frailty. Boruta is an all‐relevant features selection method based on random forest [25]. We executed the Boruta algorithm using the default settings of 500 trees (ntree = 500) and a maximum of 500 iterations (maxRuns = 500), and a verbosity level of 2 (doTrace = 2). The algorithm compared the importance of original attributes with randomly permuted copies (shadow features) to determine which variables were significantly related to frailty.

The result of the Boruta algorithm was visualized using a variable importance plot, which illustrated the relative importance of each feature. The plot would also categorize the feature into confirmed important variables, tentative (uncertain) variables, and rejected unimportant variables. Blue boxes were shown to represent shadow features.

### 2.6 Statistical Analysis

Descriptive statistics of each feature between the frailty and non‐frailty populations were first presented. Group comparisons between frail and non‐frail participants were performed using the Mann‐Whitney U test for continuous variables, given their non‐normal distributions, and the Chi‐squared test for categorical variables. Significance level (two‐tailed) was set at *p* < 0.05. A Bayesian mixed‐effects logistic regression analysis [26] was conducted to analyze the relationship between the selected features and frailty. Although initially planned to account for potential repeated participants across cohorts, subsequent analysis revealed no repeated measurements between the 2011 and 2015 cohorts. The mixed‐effects structure was retained as pre‐specified, with random intercepts for cohort effects showing minimal variance, confirming negligible between‐cohort heterogeneity and supporting the pooling of data from both waves.

We specified weakly informative priors for the fixed effects with a mean of zero and a standard deviation of 2, and a half‐Cauchy prior with a scale parameter of 1 for the random effect standard deviation. The model was fitted using four Markov Chain Monte Carlo (MCMC) chains with default settings, each running for 4000 iterations with a warm‐up period of 1000 iterations. The adapt‐delta parameter was set to 0.95 to improve sampling efficiency. Odds ratios and their 95% credible intervals (CrI) were extracted from the posterior distribution of the model parameters. Marginal effect plots were generated to illustrate the predicted probability of frailty across the range of each predictor, adjusted for other factors. Sensitivity analysis was conducted to evaluate the impact of data imputation on the Bayesian analysis. It shall be noted that the Bayesian method can handle missing data without requiring complete case deletion.

All statistical analyses were conducted using R version 4.4.1 in RStudio IDE (ver. 2024.12). Data imputation was performed using MICE via the mice package [27]. Feature selection was conducted using the Boruta package [25] and the Bayesian mixed‐effects logistic regression model was fitted using the brms package [28].

## 3 Results

### 3.1 Data Summary

The initial dataset contained 38,800 observations (17,705 observations in 2011 cohort and 21,095 observations in 2015 cohort). 1,924 participants with chronic kidney disease were screened after removing missing data. Among these participants, 634 (33%) were classified as frail. The mean age of the cohort was 63.96 years (SD 10.67, range 19 to 102), with frail individuals being significantly older than non‐frail individuals (66.08 vs. 62.92 years, *p* < 0.001). Gender distribution showed a higher proportion of frailty among females compared to males (*p* < 0.001). Frail participants had a significantly lower BMI (median: 23.00 vs. 23.45 kg/m^2^, *p* < 0.001), and smaller waist circumference compared to non‐frail participants (median: 85.20 vs. 86.50, *p* = 0.011).

Comorbidities and health problems were more prevalent in the frail group, especially, depression, rheumatism, poor vision, hypertension, and chronic lung disease. Frail participants reported poor sleep quality, with 17.2% experiencing restless sleep most or all of the time than the non‐frail individuals (*p* < 0.001). In addition, blood test results revealed significant difference between frail and non‐frail participants. Frail individuals had significantly lower creatinine, haemoglobin, and haematocrit levels but higher CRP, BUN and cystatin C levels. The data summaries are shown in Table 1, Table 2, and Table S1.

**Table 1:** Demographic and clinical characteristics (categorical variables) of Chronic kidney disease patients in the CHARLS cohort.

Variable, *N* (%)			Overall	Non‐frailty	Frailty	*p* value
*N* (Total)			1924	1290 (67)	634 (33)	
*N* (2011CHARLS)			762	489 (64)	273 (36)	
*N* (2015CHARLS)			1162	801 (69)	361 (31)	
Gender	(Female)		785 (40.8)	462 (35.8)	323 (50.9)	< 0.001
	(Male)		1139 (59.2)	828 (64.2)	311 (49.1)	
Alcohol consumption			653 (34.0)	503 (39.1)	150 (23.7)	< 0.001
Sleep quality		( = 0)	790 (43.0)	609 (49.3)	181 (30.0)	< 0.001
		( = 1)	252 (13.7)	177 (14.3)	75 (12.4)	
		( = 2)	288 (15.7)	177 (14.3)	111 (18.4)	
		( = 3)	509 (27.7)	272 (22.0)	237 (39.2)	
Smoking			437 (32.0)	292 (32.8)	145 (30.6)	0.446
Social activity			1060 (55.1)	753 (58.4)	307 (48.4)	< 0.001
Cancer			31 (1.6)	18 (1.4)	13 (2.1)	0.382
Chronic lung diseases			338 (17.6)	188 (14.6)	150 (23.7)	< 0.001
Depression			801 (46.5)	413 (35.2)	388 (70.7)	< 0.001
Diabetes			210 (11.0)	116 (9.0)	94 (14.9)	< 0.001
Digestive disease			657 (34.2)	398 (30.9)	259 (40.9)	< 0.001
Dyslipidaemia			323 (16.9)	195 (15.2)	128 (20.4)	0.005
Hypertension			771 (40.2)	475 (36.9)	296 (46.8)	< 0.001
Pain			1496 (77.8)	973 (75.4)	523 (82.5)	0.001
Poor hearing			355 (18.8)	198 (15.6)	157 (25.4)	< 0.001
Poor vision			765 (41.2)	433 (34.8)	332 (54.5)	< 0.001
Psychiatric problems			35 (1.8)	15 (1.2)	20 (3.2)	0.004
Stroke			88 (4.6)	40 (3.1)	48 (7.6)	< 0.001
Rheumatism			886 (46.1)	537 (41.7)	349 (55.1)	< 0.001

**Table 2:** Distribution of continuous variables among chronic kidney disease patients in the CHARLS study.

Variable	Total	Non‐frailty	Frailty	^ *p‐*value
Age	64.00 [56.00, 72.00]	63.00 [55.00, 70.00]	66.00 [58.00, 74.00]	< 0.001
BMI	23.30 [20.80, 26.20]	23.45 [21.22, 26.30]	23.00 [19.60, 25.80]	< 0.001
Waist ccm	86.00 [78.50, 93.40]	86.50 [79.15, 93.40]	85.20 [76.05, 93.20]	0.011
BUN (mg/dL)	16.50 [13.40, 20.40]	16.35 [13.40, 19.90]	16.80 [13.43, 21.10]	0.014
Creatinine (mg/dL)	0.95 [0.74, 1.33]	0.95 [0.75, 1.34]	0.93 [0.71, 1.32]	0.063
CRP (mg/L)	1.48 [0.78, 3.00]	1.38 [0.72, 2.70]	1.70 [0.80, 4.36]	< 0.001
Cystatin C (mg/L)	0.99 [0.82, 1.27]	0.97 [0.80, 1.22]	1.05 [0.84, 1.41]	< 0.001
Glucose (mg/dL)	99.10 [90.85, 110.70]	98.70 [90.10, 110.15]	99.10 [91.60, 111.70]	0.562
Gly. Hb (%)	5.60 [5.20, 6.00]	5.60 [5.20, 6.00]	5.60 [5.20, 6.10]	0.462
Hb (g/dL)	13.70 [12.50, 15.10]	13.90 [12.70, 15.30]	13.30 [12.10, 14.70]	< 0.001
Hc (%)	41.10 [37.40, 45.00]	41.60 [37.80, 45.40]	40.35 [36.50, 44.00]	< 0.001
HDL (mg/dL)	49.40 [41.30, 58.70]	49.40 [41.40, 58.00]	49.45 [40.90, 59.10]	0.872
LDL (mg/dL)	104.30 [85.30, 125.60]	103.90 [85.70, 125.30]	105.85 [85.30, 126.38]	0.743
MCV (fL)	92.20 [87.70, 96.60]	92.00 [87.70, 96.50]	92.50 [87.82, 96.90]	0.151
Platelets (10^9^/L)	191.00 [149.75, 243.00]	190.00 [150.93, 238.75]	193.00 [148.25, 249.00]	0.402
TC (mg/dL)	182.50 [160.80, 208.50]	182.15 [161.65, 207.28]	183.10 [159.10, 211.30]	0.684
TG (mg/dL)	108.90 [79.70, 163.70]	108.00 [78.80, 161.90]	112.40 [81.40, 165.50]	0.255
Uric acid (mg/dL)	5.10 [4.10, 6.40]	5.10 [4.10, 6.33]	5.03 [4.00, 6.47]	0.350
WBC (k/μL)	5.90 [4.90, 7.10]	5.90 [4.90, 7.10]	6.00 [4.90, 7.40]	0.128

*Note:* All *p* values are calculated based on non‐parametric tests. Data shown are median with interquartile.

Abbreviations: BMI, body mass index; BUN, blood urea nitrogen; ccm, circumference; CRP, C‐reactive protein; Gly., Glycated; Hb, haemoglobin; Hc, Haematocrit; HDL, high‐density lipoprotein; LDL, low‐density lipoprotein; MCV, mean corpuscular volume; TC, total cholesterol; TG, triglyceride; WBC, white blood cell counts.

### 3.2 Feature Selection

**Figure 1:**
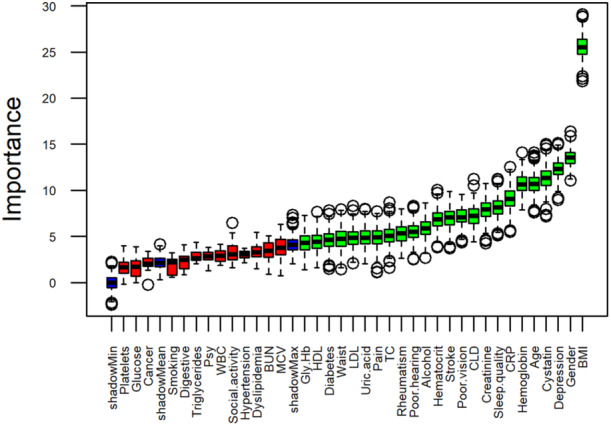
Variable Importance plot of the Boruta algorithm. Green boxplots represent confirmed important variables, red boxplots represent rejected variables, and blue boxplots represent shadow features. BMI, Body Mass Index; BUN, Blood Urea Nitrogen; CLD, Chronic Lung Disease; CRP, C‐reactive Protein; Gly. Hb, Glycated Haemoglobin; HDL, High‐density Lipoprotein; LDL, Low‐density Lipoprotein; MCV, Mean Corpuscular Volume; Psy, Psychiatric Problems; TC, Total Cholesterol; WBC, White Blood Cell Count.

**Figure 2:**
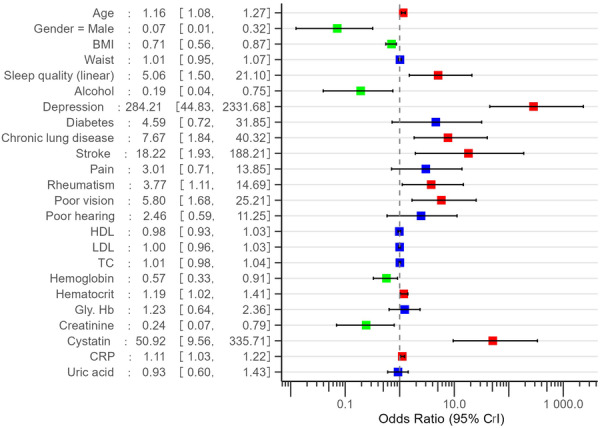
Odds ratio of predictors of frailty among chronic kidney disease, adjusted for other factors. Red represents significant association with OR > 1; green represents significant association with OR < 1; blue represents non‐significant association with OR 95% CI across 1. BMI, body mass index; CRP, c‐reactive protein; Gly. Hb, glycated haemoglobin; HDL, high‐density lipoprotein; LDL, low‐density lipoprotein; TC, total cholesterol.

The variable importance plot, illustrated in Figure 1, provides a visual representation of the relative importance of each feature. From the initial set of 37 features, the algorithm identified 24 as important and selected them for further analysis, while rejecting the remaining 13. Notably, BMI emerged as the feature with the highest importance. The selected 24 features are shown in the model in Figure 2 and Table S3.

### 3.3 Bayesian Model

The Bayesian mixed‐effect logistic regression model revealed several significant predictors of frailty in chronic kidney disease patients (Figure 2). Depression emerged as the strongest predictor, with patients having depression showing dramatically higher odds of frailty (adjusted OR 289.49, 95%CrI 47.24–2284.82). Other significant comorbidities associated with increased frailty risk included stroke (adjusted OR 18.04, 95%CrI 1.94– 183.99), chronic lung disease (adjusted OR 7.96, 95%CrI 1.84–39.44), and rheumatism (adjusted OR 3.84 95%CrI 1.12–15.14). Poor sleep quality and poor vision were both associated with approximately 5 times higher odds of frailty (adjusted OR 5.12, 95%CrI 1.53–21.13 and adjusted OR 5.84, 95%CrI 1.68–25.25, respectively).

Among the blood test data, elevated cystatin C levels were strongly associated with frailty (adjusted OR 52.46, 95%CrI 9.51–356.12). CRP showed a modest positive association (adjusted OR 1.11, 95%CrI 1.03–1.22). Conversely, for every 1 unit increase in creatinine, the odds of frailty decreased by 76% (adjusted OR 0.24, 95%CrI 0.07–0.81). Each unit increase in haemoglobin was associated with 42% reduction in odds of frailty (adjusted OR 0.58, 95%CrI 0.34–0.91).

Besides, age was positively associated with frailty (adjusted OR 1.16, 95%CrI 1.08–1.27), while higher BMI was associated with decreased frailty risk (adjusted OR 0.71, 95%CrI 0.56–0.87). Male (OR 0.07, 95%CrI 0.01–0.32) and alcohol consumption (adjusted OR 0.19, 95%CrI 0.04–0.74) were associated with lower odds of frailty. Figure S2 shows the marginal effect plots of the Bayesian model.

### 3.4 Sensitivity Test

A sensitivity analysis using complete case analysis (no imputation) showed results largely consistent with our main analysis, with all associations maintaining the same direction (Table S4). However, some variables that were significant in the main analysis (sleep quality, stroke, and creatinine) became non‐significant, while other strong predictors (depression, chronic lung disease, cystatin C) remained significant with similar effect sizes. Notably, the odds ratio for poor sleep quality (linear term) changed appreciably after data imputation. Overall, these findings indicate that our primary conclusions are generally robust to imputation methods.

## 4 Discussion

Frailty is a multifaceted syndrome that significantly impacts health outcomes in older adults particularly those with CKD [10]. The intersection of frailty and CKD is critical, as frailty not only exacerbates the progression of kidney disease but also increases the risk of adverse outcomes, such as hospitalization, cardiovascular events, and mortality [5, 29]. This study aimed to understand potential risk factor for frailty among CKD patients using dataset from CHARLS. There remains a scarcity of research focused on estimating frailty risk among individuals with CKD. One study has attempted to predict “cognitive frailty” by albumin levels and sociodemographic factors, such as education level and perceived social support [30].

While comorbidities, such as stroke, chronic lung disease, and rheumatism, might induce frailty in individuals [31, 32, 33], the emergence of depression as the strongest predictor is particularly noteworthy. The adjusted odds ratio for depression substantially exceeds that of other factors. Depression can lead to reduced physical activity, poor nutritional intake, and social isolation, all of which are known risk factors for frailty [34]. Additionally, depression and frailty may share common pathophysiological pathways, such as chronic inflammation and dysregulation of the hypothalamic‐pituitary‐adrenal axis [35, 36]. Furthermore, their relationship with CKD in the inflammation pathway is bidirectional, forming a vicious cycle that implicates progression [37, 38]. On the other hand, sleep quality and vision impairment emerged as significant potential contributors to frailty. Poor sleep quality may exacerbate fatigue and reduce physical function [39], while poor vision, prevalent among CKD [40], can lead to reduced mobility, increased risk of falls, and social isolation [41, 42], all of which can contribute to the further development of frailty among CKD patients.

The blood test results, particularly elevated levels of cystatin C and CRP, along with decreased haemoglobin levels, reflected the increased severity of CKD [43, 44, 45] and thus its association with frailty development. CKD patients with lower eGFR had significantly higher odds of frailty [46]. However, the relationship between creatinine levels and frailty presented a counterintuitive finding. We found that increases in creatinine levels reduced the odds of frailty. This unexpected finding requires cautious interpretation. While higher creatinine levels typically indicate worse kidney function [47], the relationship between creatinine and frailty in CKD patients is complex and may be influenced by multiple factors beyond simple muscle mass association [48]. It was suggested that muscle quality, including factor such as intramuscular fat infiltration and myosteatosis, may be more relevant to frailty development than muscle mass alone [49, 50]. Besides, CKD patients often adhere to a strict protein diet [51], which might reduce muscle mass. In contrast, cystatin C levels are less affected by diet [52]. Our findings regarding age, gender, BMI, and waist circumference further support this notion related to muscle mass. Advanced age is associated with a higher risk of sarcopenia, while being male and having a higher BMI may reflect greater muscle mass [53, 54]. This subtle tension between the negative impact of declining kidney function and the potential protective effect of preserved muscle mass in frailty deserves further investigation.

There were some limitations in this study. Firstly, this study adopted the CHARLS dataset, that focused on the Chinese population aged 45 and older and their households, which may limit the generalizability of our findings. Second, missing data is a common issue in large‐scale epidemiological surveys [55]. In our study, we encountered a substantial amount of missing data. For participants lacking basic information and blood test data, we had to discard the data, potentially introduce exclusion bias. For other missing variables, we implemented imputation techniques, assuming the data were missing at random (MAR) [18]. While this approach is widely accepted, the extent of missing data in our study raises concerns about the validity of the MAR assumption. Our sensitivity analysis further revealed that the measure of poor sleep quality varied after imputation, suggesting that the imputation process may influence the stability of this self‐reported variable. Specifically, sleep quality in CHARLS was assessed subjectively based on the frequency of restless sleep across four categories (rare/none, some/little, occasional, most/all nights), which could introduce recall or reporting bias. Additionally, our frailty assessment was limited by incomplete availability of all criteria for some participants, necessitating proportional adjustment of the frailty threshold. This adaptation may have affected the accuracy of frailty classification compared to the standard phenotype definition. Third, health conditions and comorbidities were based on self‐reports, which may be subject to recall bias or misreporting, while we also observe some inconsistencies between the questionnaires used in the two cohorts, which may have introduced measurement bias.

Fourth, our study design was cross‐sectional rather than longitudinal, with no repeated measurements within or between cohorts. While we initially employed a mixed‐effects framework anticipating potential repeated participants, the analysis revealed independent samples across the 2011 and 2015 waves. This precludes examination of temporal changes in frailty status or causal relationships between risk factors and frailty development over time. Future longitudinal studies with multiple follow‐up points are needed to establish temporal relationships and examine frailty progression in CKD patients. Moreover, blood test data were only available for approximately half of the full population and limited to these two cohort years. This limitation in data availability, combined with missing data issues, made it unfeasible to conduct more comprehensive longitudinal or survival analyses. This restriction in our analytical approach may have prevented us from capturing the full temporal dynamics of frailty development in CKD patients. Furthermore, our study employed a modified Fried frailty phenotype [11, 19, 56]. While this approach may not capture the full precision of standardized grip strength and gait speed measurements, it maintains the multidimensional conceptual framework of frailty assessment [19, 57]. The exclusion of grip strength measurements represents a limitation, as grip strength is considered one of the most objective and reliable frailty indicators [11, 56]. However, the substantial missing data and measurement inconsistencies in CHARLS would have introduced greater bias than the use of self‐reported weakness as a proxy. Future studies with standardized grip strength protocols across all survey waves would strengthen frailty classification accuracy.

Lastly, we relied on creatinine‐based CKD‐EPI equation for CKD classification. While the CKD‐EPI equation is one of the most representable methods, this method may have led to misclassification of CKD status due to factors unrelated to glomerular filtration [58, 59], especially those with altered muscle mass, potentially affecting our results. Future research should incorporate cystatin C‐based or combined creatinine‐cystatin C eGFR equations for CKD classification, which are less affected by covariates [60]. The availability of both creatinine and cystatin C measurements would allow for comparison of different eGFR equations and potentially improve the accuracy of CKD classification and its relationship with frailty.

## 5 Conclusions

This study provides valuable insights into the potential contributing factors of frailty among chronic kidney disease patients using the CHARLS dataset. Depression emerged as the strongest predictor, highlighting the critical importance of mental health in CKD patients. Comorbidities such as stroke, chronic lung disease, and rheumatism significantly increased frailty risk. Vision impairment also strongly contributed markedly to frailty risk. Poor sleep quality, though associated with higher frailty odds, showed some instability after data imputation and should therefore be interpreted with caution. Blood markers, particularly elevated cystatin C and C‐reactive protein levels, were linked to frailty, reflecting the impact of kidney dysfunction and systemic inflammation. Interestingly, higher creatinine levels were associated with lower frailty risk, potentially due to its relationship with muscle mass.

## Author Contributions

**Jiao Wang:** investigation, data curation, software, formal analysis, writing – original draft. **Quan Wang:** investigation, writing – original draft, software, formal analysis, data curation. **Zian Zhao:** investigation, validation, visualization. **Minxin Chen:** investigation, validation, visualization. **Dexter Siu‐Hong Wong:** writing – review and editing, validation, supervision. **James Chung‐Wai Cheung:** conceptualization, methodology, writing – review and editing, supervision, project administration. **Duo Wai‐Chi Wong:** conceptualization, methodology, writing – review and editing, supervision, project administration, funding acquisition.

## Funding

The authors received no specific funding for this work.

## Conflicts of Interest

The authors declare no conflicts of interest.

## Transparency Statement

The corresponding author (James Chung‐Wai Cheung and Duo Wai‐Chi Wong) affirms that this manuscript is an honest, accurate, and transparent account of the study being reported; that no important aspects of the study have been omitted; and that any discrepancies from the study as planned (and, if relevant, registered) have been explained. All authors have read and approved the final version of the manuscript.

## Supporting information


**Figure S1:** Study flowchart showing participant selection and analysis workflow for frailty risk factors in chronic kidney disease patients from the CHARLS cohorts. **Figure S2:** Marginal effect plot of Bayesian model. **Table S1:** Distribution of Continuous Variables (Mean, SD, Median, IQR) Among Chronic Kidney Disease Patients in the CHARLS Study. **Table S2:** Number of imputed missing data after exclusion criteria. **Table S3:** Results of Bayesian mixed‐effect logistic regression model. **Table S4:** Sensitivity analysis on the Bayesian mixed‐effect logistic regression model without data imputation.

## Data Availability

Data were obtained from CHARLS and are available from https://charls.pku.edu.cn/.
