# Supplementary material for: Bayesian Analysis of Frailty Risk Factors in Chronic Kidney Disease: A Nationwide Cross‐Sectional Survey
Source: Health Sci Rep. 2025 Dec 29;9(1):e71698. doi: 10.1002/hsr2.71698 (PMC12745903; doi:10.1002/hsr2.71698)
Supplement: Supplementary file 1 — Figure S1: Study flowchart showing participant selection and analysis workflow for frailty risk factors in chronic kidney disease patients from the CHARLS cohorts. Figure S2: Marginal effect plot of Bayesian model. Table S1: Distribution of Continuous Variables (Mean, SD, Median, IQR) Among Chronic Kidney Disease Patients in the CHARLS Study. Table S2: Number of imputed missing data after exclusion criteria. Table S3: Results of Bayesian mixed‐effect logistic regression model. Table S4: Sensitivity analysis on the Bayesian mixed‐effect logistic regression model without data imputation. [file HSR2-9-e71698-s001.pdf]

Figure S1. Study flowchart showing participant selection and analysis workflow for frailty risk factors in chronic kidney disease patients from the CHARLS cohorts.

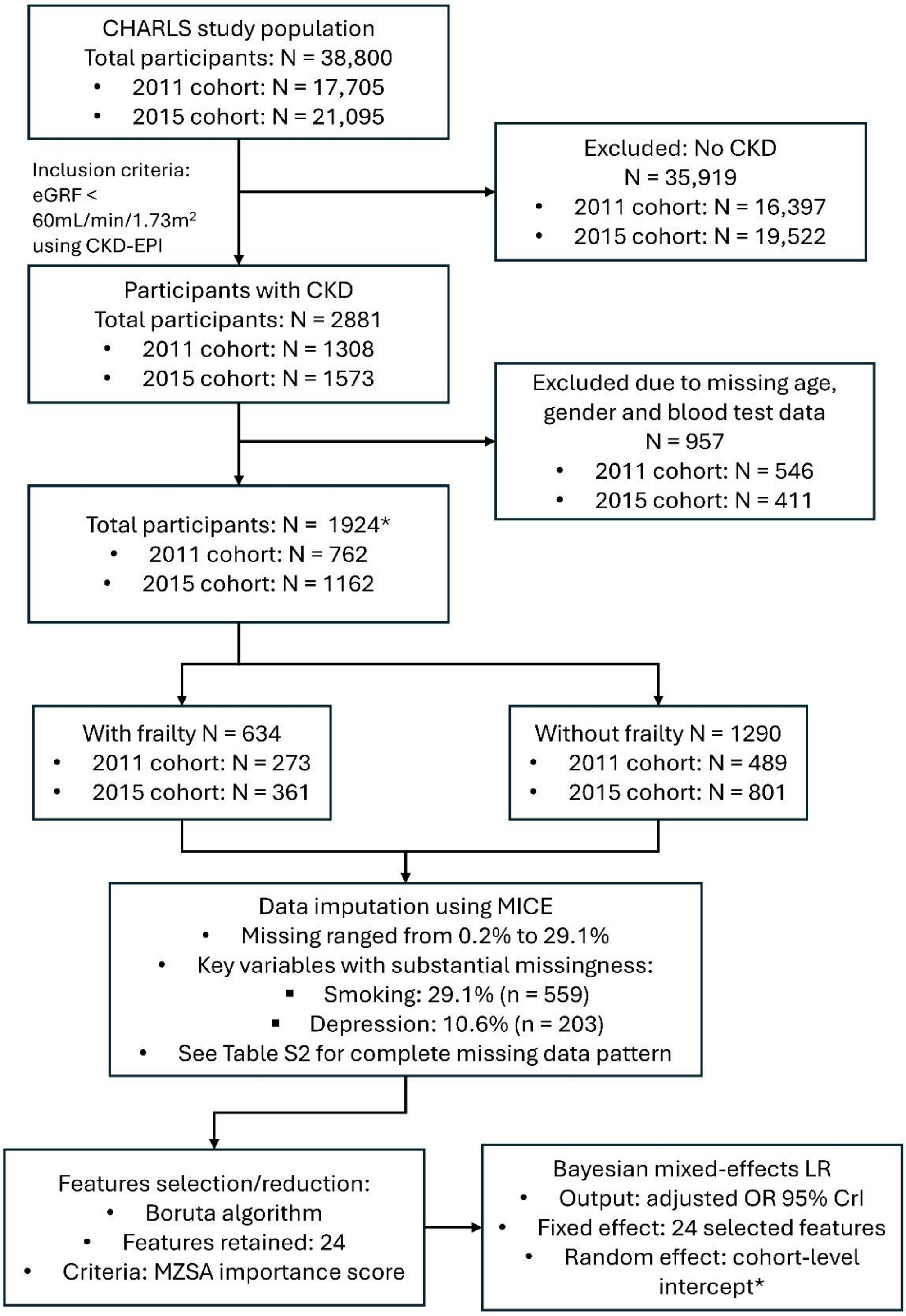

CrI: credible interval; CKD: chronic kidney disease; CKD-EPI: Collaborative Epidemiological Study of CKD equation; MICE: multivariate imputation by chained equations; MZSA: Maximum Z-score among shadow attributes; OR: odds ratio. *No repeated measurement identified across cohorts. Mixed effects model with minimal random effect variance.

Figure S2. Marginal effect plot of Bayesian model

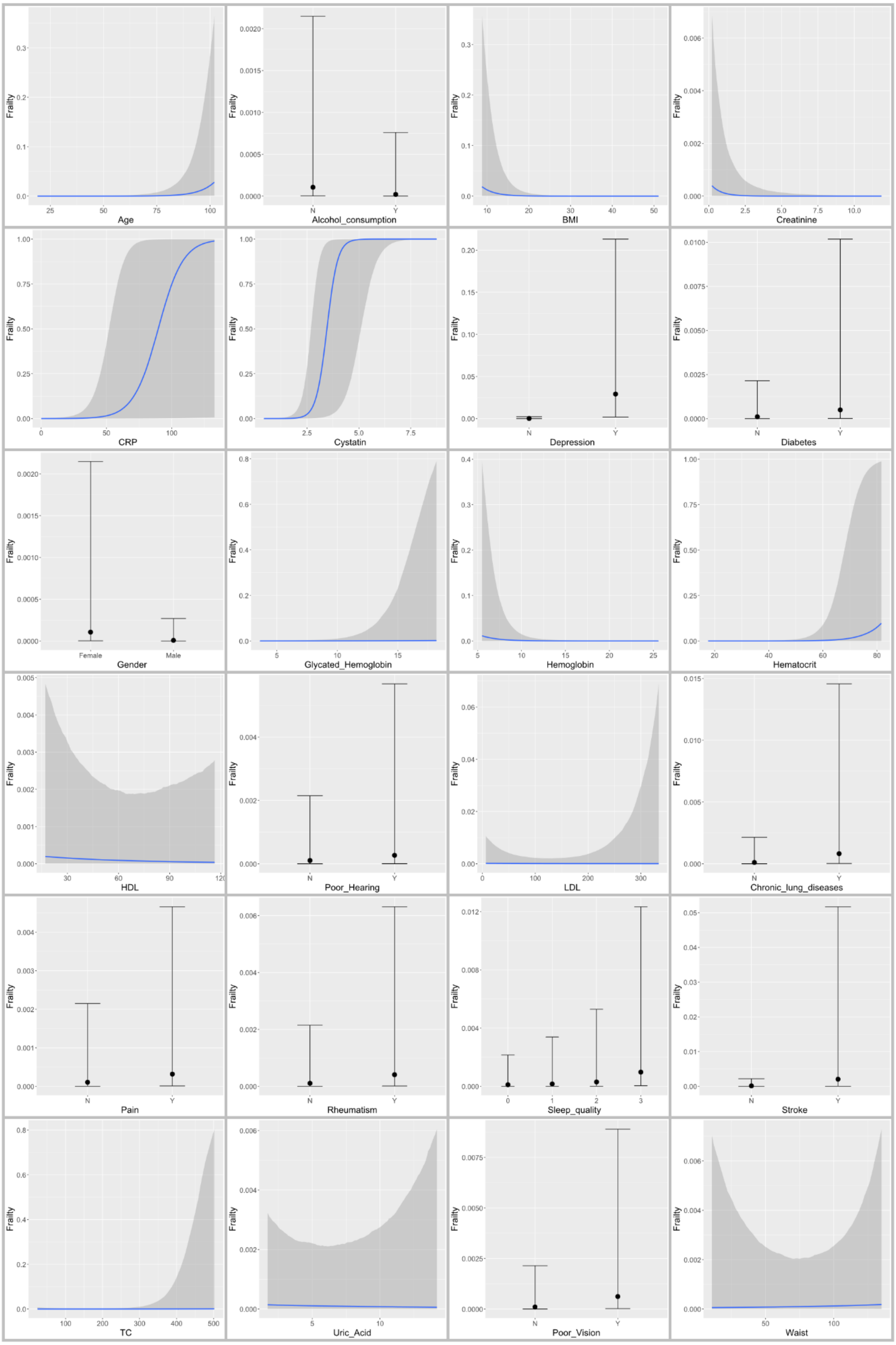

Table S1. Distribution of Continuous Variables (Mean, SD, Median, IQR) Among Chronic Kidney Disease Patients in the CHARLS Study

Variable	Total		Non-Frailty		Frailty		^p-value
	Mean[SD]	Median [IQR]	Mean[SD]	Median [IQR]	Mean[SD]	Median [IQR]	
Age	63.96 [10.67]	64.00 [56.00, 72.00]	62.92 [10.45]	63.00 [55.00, 70.00]	66.08 [10.79]	66.00 [58.00, 74.00]	<0.001
BMI	23.65 [4.07]	23.30 [20.80, 26.20]	23.94 [3.82]	23.45 [21.22, 26.30]	23.04 [4.49]	23.00 [19.60, 25.80]	<0.001
Waist ccm	85.22 [14.19]	86.00 [78.50, 93.40]	85.79 [13.67]	86.50 [79.15, 93.40]	83.98 [15.20]	85.20 [76.05, 93.20]	0.011
CRP (mg/L)	3.74 [8.92]	1.48 [0.78, 3.00]	3.09 [6.72]	1.38 [0.72, 2.70]	5.05 [12.13]	1.70 [0.80, 4.36]	<0.001
Creatinine (mg/dL)	1.12 [0.69]	0.95 [0.74, 1.33]	1.11 [0.55]	0.95 [0.75, 1.34]	1.14 [0.92]	0.93 [0.71, 1.32]	0.063
WBC (k/μL)	6.21 [1.94]	5.90 [4.90, 7.10]	6.14 [1.82]	5.90 [4.90, 7.10]	6.33 [2.14]	6.00 [4.90, 7.40]	0.128
Hb (g/dL)	13.82 [2.13]	13.70 [12.50, 15.10]	14.03 [2.09]	13.90 [12.70, 15.30]	13.40 [2.15]	13.30 [12.10, 14.70]	<0.001
Hc (%)	41.17 [6.11]	41.10 [37.40, 45.00]	41.58 [5.87]	41.60 [37.80, 45.40]	40.34 [6.50]	40.35 [36.50, 44.00]	<0.001
MCV (fL)	91.27 [8.64]	92.20 [87.70, 96.60]	91.13 [8.36]	92.00 [87.70, 96.50]	91.56 [9.18]	92.50 [87.82, 96.90]	0.151
Platelets (10^9/L)	198.33 [76.02]	191.00 [149.75, 243.00]	196.98 [74.44]	190.00 [150.93, 238.75]	201.07 [79.11]	193.00 [148.25, 249.00]	0.402
TG (mg/dL)	138.88 [94.64]	108.90 [79.70, 163.70]	138.75 [97.54]	108.00 [78.80, 161.90]	139.13 [88.51]	112.40 [81.40, 165.50]	0.255
BUN (mg/dL)	17.72 [7.14]	16.50 [13.40, 20.40]	17.31 [6.14]	16.35 [13.40, 19.90]	18.57 [8.78]	16.80 [13.43, 21.10]	0.014
Uric Acid (mg/dL)	5.36 [1.73]	5.10 [4.10, 6.40]	5.37 [1.70]	5.10 [4.10, 6.33]	5.32 [1.79]	5.03 [4.00, 6.47]	0.350
Gly. Hb (%)	5.74 [1.05]	5.60 [5.20, 6.00]	5.72 [1.01]	5.60 [5.20, 6.00]	5.79 [1.13]	5.60 [5.20, 6.10]	0.462
HDL (mg/dL)	50.78 [13.53]	49.40 [41.30, 58.70]	50.94 [13.43]	49.40 [41.40, 58.00]	50.45 [13.72]	49.45 [40.90, 59.10]	0.872
LDL (mg/dL)	107.84 [33.81]	104.30 [85.30, 125.60]	107.79 [33.42]	103.90 [85.70, 125.30]	107.95 [34.62]	105.85 [85.30, 126.38]	0.743
TC (mg/dL)	187.50 [41.14]	182.50 [160.80, 208.50]	187.51 [40.92]	182.15 [161.65, 207.28]	187.48 [41.62]	183.10 [159.10, 211.30]	0.684
Glucose (mg/dL)	108.05 [38.74]	99.10 [90.85, 110.70]	107.47 [37.19]	98.70 [90.10, 110.15]	109.22 [41.74]	99.10 [91.60, 111.70]	0.562
Cystatin C (mg/L)	1.13 [0.55]	0.99 [0.82, 1.27]	1.07 [0.43]	0.97 [0.80, 1.22]	1.24 [0.73]	1.05 [0.84, 1.41]	<0.001

^all p-values are calculated based on non-parametric tests.

BMI: body mass index; ccm: circumference; CRP: C-reactive protein; WBC: white blood cell counts; Hb: haemoglobin; Hc: Haematocrit; MCV: mean corpuscular volume; BUN: blood urea nitrogen; TG: triglyceride; Gly.: Glycated; HDL: high-density lipoprotein; LDL: low-density lipoprotein; TC: total cholesterol.

Table S2. Number of imputed missing data after exclusion criteria

Variable, N (%)	Counts (2015 Cohort)	Counts (2011 Cohort)	Counts (Total)
Body height	31(1.6)	124(6.4)	155(8.1)
Body weight	27(1.4)	120(6.2)	147(7.6)
Smoking	557(29.0)	2(0.1)	559(29.1)
Alcohol Consumption	2(0.1)	2(0.1)	4(0.2)
Sleep Quality	45(2.3)	40(2.1)	85(4.4)
Waist circumference	25(1.3)	118(6.1)	143(7.4)
Hypertension	0(0.0)	5(0.3)	5(0.3)
Dyslipidaemia	0(0.0)	17(0.9)	17(0.9)
Diabetes	0(0.0)	7(0.4)	7(0.4)
Cancer	0(0.0)	3(0.2)	3(0.2)
Chronic Lung diseases	0(0.0)	3(0.2)	3(0.2)
Liver disease	0(0.0)	5(0.3)	5(0.3)
Stroke	0(0.0)	4(0.2)	4(0.2)
Digestive disease	0(0.0)	3(0.2)	3(0.2)
Psychiatric problems	0(0.0)	5(0.3)	5(0.3)
Rheumatism	0(0.0)	4(0.2)	4(0.2)
Poor Hearing	37(1.9)	3(0.2)	40(2.1)
Poor Vision	40(2.1)	29(1.5)	69(3.6)
Depression	126(6.5)	77(4.0)	203(10.6)

Table S3. Results of Bayesian mixed-effect logistic regression model.

Predictors	Odds Ratio	Lower CrI	Upper CrI	Beta	Beta (Lower CrI)	Beta (Upper CrI)
Intercept	0.00	0.00	0.01	-13.67	-24.22	-4.78
Gender = Male	0.07	0.01	0.32	-2.65	-4.34	-1.14
Age	1.16	1.08	1.27	0.15	0.07	0.24
BMI	0.71	0.56	0.87	-0.35	-0.60	-0.14
Waist ccm	1.01	0.95	1.07	0.01	-0.05	0.07
Alcohol consumption	0.19	0.04	0.74	-1.66	-3.22	-0.30
Sleep quality (linear)	5.12	1.53	21.13	1.64	0.40	3.09
Sleep quality (quadratic)	1.54	0.43	5.68	0.42	-0.85	1.72
Sleep quality (cubic)	1.05	0.26	4.27	0.07	-1.36	1.53
Diabetes	4.54	0.74	32.38	1.51	-0.32	3.46
Chronic lung disease	7.96	1.84	39.44	2.08	0.64	3.72
Stroke	18.04	1.94	183.93	2.91	0.65	5.28
Rheumatism	3.84	1.12	15.14	1.34	0.15	2.73
Pain	2.96	0.70	14.03	1.09	-0.36	2.67
Depression	289.49	47.24	2285.82	5.69	3.86	7.81
CRP	1.11	1.03	1.22	0.11	0.03	0.20
Creatinine	0.24	0.07	0.81	-1.41	-2.68	-0.18
Haemoglobin	0.57	0.34	0.91	-0.56	-1.10	-0.09
Haematocrit	1.19	1.02	1.40	0.17	0.02	0.34
Uric Acid	0.93	0.60	1.42	-0.07	-0.50	0.35
Glycated Haemoglobin	1.23	0.66	2.32	0.22	-0.41	0.86
HDL	0.98	0.93	1.03	-0.02	-0.07	0.03
LDL	1.00	0.96	1.03	0.00	-0.04	0.03
TC	1.01	0.98	1.03	0.01	-0.02	0.03
Cystatin	52.46	9.51	356.12	3.94	2.23	5.85
Poor Hearing	2.45	0.58	11.84	0.91	-0.51	2.48
Poor Vision	5.84	1.68	25.25	1.78	0.53	3.26

BMI: body mass index; ccm: circumference; CRP: C-reactive protein; HDL: high-density lipoprotein; LDL: low-density lipoprotein; TC: total cholesterol.

Table S4. Sensitivity analysis on the Bayesian mixed-effect logistic regression model without data imputation.

Predictors	Odds Ratio	Lower CrI	Upper CrI	Beta	Beta (Lower CrI)	Beta (Upper CrI)
Intercept	0.00	0.00	0.01	-14.76	-26.29	-4.47
Gender = Male	0.07	0.01	0.37	-2.64	-4.52	-0.99
Age	1.17	1.07	1.29	0.15	0.07	0.26
BMI	0.72	0.55	0.91	-0.33	-0.61	-0.09
Waist ccm	1.01	0.95	1.08	0.01	-0.05	0.08
Alcohol consumption	0.22	0.04	0.96	-1.53	-3.22	-0.04
Sleep quality (linear)	3.22	0.87	14.47	1.17	-0.14	2.67
Sleep quality (quadratic)	1.57	0.37	6.74	0.45	-0.99	1.91
Sleep quality (cubic)	1.01	0.21	4.91	0.01	-1.56	1.59
Diabetes	3.31	0.47	27.65	1.20	-0.76	3.32
Chronic lung disease	8.22	1.74	45.89	2.11	0.55	3.83
Stroke	6.64	0.55	86.45	1.89	-0.60	4.46
Rheumatism	4.25	1.12	19.54	1.45	0.12	2.97
Pain	2.50	0.49	14.25	0.92	-0.72	2.66
Depression	558.70	71.19	5428.91	6.33	4.27	8.60
CRP	1.09	1.00	1.19	0.08	0.00	0.18
Creatinine	0.30	0.08	1.06	-1.21	-2.55	0.06
Haemoglobin	0.50	0.27	0.85	-0.69	-1.30	-0.16
Haematocrit	1.22	1.03	1.48	0.20	0.03	0.39
Uric Acid	0.97	0.60	1.61	-0.03	-0.52	0.47
Glycated Haemoglobin	1.36	0.68	2.89	0.31	-0.39	1.06
HDL	0.99	0.94	1.06	-0.01	-0.07	0.05
LDL	1.00	0.97	1.04	0.00	-0.03	0.04
TC	1.00	0.97	1.03	0.00	-0.04	0.03
Cystatin	29.50	4.87	205.16	3.38	1.58	5.32
Poor Hearing	2.64	0.54	14.71	0.97	-0.61	2.69
Poor Vision	5.77	1.48	28.14	1.75	0.39	3.34

BMI: body mass index; ccm: circumference; CRP: C-reactive protein; HDL: high-density lipoprotein; LDL: low-density lipoprotein; TC: total cholesterol.
